# The complexities of respiratory chain biogenesis and maintenance in yeast mitochondria

**DOI:** 10.1002/pro.70545

**Published:** 2026-03-25

**Authors:** Andreas Carlström, Carmela Vazquez‐Calvo, Martin Ott

**Affiliations:** ^1^ Department of Medical Biochemistry and Cell Biology Institute of Biomedicine, University of Gothenburg Gothenburg Sweden

**Keywords:** assembly, biogenesis, complex III, mitochondria, OXPHOS, proteostasis, quality control, regulation, respiratory chain, translation

## Abstract

Mitochondrial oxidative phosphorylation is the most efficient way of energy conversion for eukaryotic cells. It is executed by a series of high‐molecular weight enzyme complexes in the inner mitochondrial membrane that were acquired during endosymbiosis at the root of eukaryotic evolution. Biogenesis of this machinery depends not only on nuclear gene expression and protein import, but also on an organelle‐specific system to express a handful of proteins encoded in mitochondrial DNA. These two genetic systems cooperate for the biogenesis and maintenance of oxidative phosphorylation complexes. Here, we use the respiratory chain complex III as an example to highlight the complexities of this process. Specifically, we will describe the intricate mechanisms by which respiratory chain complexes are assembled, how the two genetic systems are coordinated and how biogenesis and function are physically separated within the inner mitochondrial membrane. To do so, we will primarily discuss findings from baker's yeast, where a wealth of recent data revealed exciting insights into these processes.

## 1 THE OXPHOS SYSTEM AND ITS EVOLUTIONARY PAST

Mitochondria are essential organelles of eukaryotic cells. Their acquisition through endosymbiosis at the root of eukaryotic evolution laid the foundation for the development of highly complex cellular organizations (Sagan, 1967), which, among others, resulted in the generation of multicellular life forms like fungi, plants, and animals. One of the main advantages of endosymbiosis was that the resultant cell would get access to the highly versatile process of oxidative phosphorylation, which in turn gave the cell an excess of ATP to develop complex levels of gene expression. Moreover, compartmentalization made cells capable of achieving complex biochemical pathways occurring in specialized organelles. Most genetic material found in the mitochondrial ancestor's genome has been transferred to the nuclear DNA where its expression is regulated through transcription‐factor dependent mechanisms. Mitochondria‐destined nuclear‐encoded proteins are synthesized in the cytosol and carry signals that allow their import into mitochondria through sets of dedicated import machineries (Araiso et al., 2022; Busch et al., 2023; Grevel et al., 2019; Herrmann & Bykov, 2023; Jain et al., 2025). Once inside mitochondria, these proteins mature, a process often requiring the proteolytic removal of import signals, and the subsequent folding and assembly, which can be supported by general or dedicated chaperones (Ghifari et al., 2025; Grevel et al., 2019).

**FIGURE 1:**
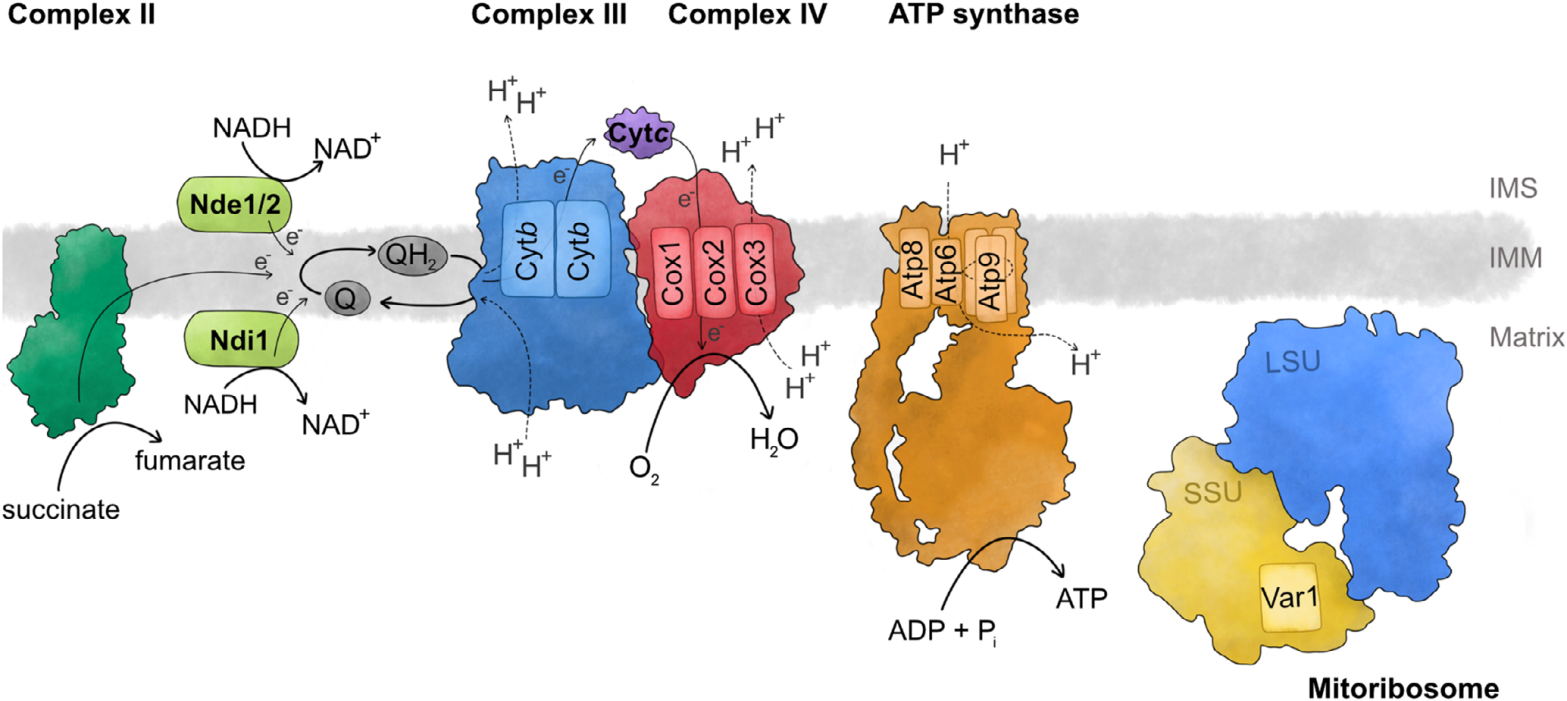
Mitochondrial encoded proteins and OXPHOS complexes. The mitochondrial oxidative phosphorylation system of yeast consists of four coupled enzymes that connect electron transfer to the final electron acceptor oxygen with the establishment of a proton‐driven membrane potential that powers the ATP synthase. Respiratory complexes III and IV and ATP synthase consist of subunits encoded in either the nuclear or the mitochondrial DNA. Also, the mitochondrial ribosome (mitoribosome) contains one mitochondrial encoded protein in the small subunit (SSU), in addition to the catalytic ribosomal RNA in both the small (SSU) and large subunits (LSU). IMM, inner mitochondrial membrane; IMS, intermembrane space.

The oxidative phosphorylation system (OXPHOS) was presumably key for endosymbiosis and eukaryotic evolution to occur. Compared with substrate‐level phosphorylation during glycolysis, oxidative phosphorylation gains substantially higher amounts of ATP, explaining why the metabolism of cells with a high energy demand typically requires mitochondrial energy conversion. Like in bacterial systems, mitochondrial OXPHOS resides in the inner membrane and transfers electrons from reduced metabolites to the final electron acceptor, molecular oxygen. During electron transfer, a proton‐motive force is established through the extrusion of protons from the mitochondrial matrix to the intermembrane space. To do so, a series of high‐molecular weight complexes accept or donate electrons from mobile electron carriers or metabolites and connect the electron transfer to proton translocation (Figure 1). In the mitochondrial respiratory chain, electrons derived from NADH or succinate are first transferred to coenzyme Q by NADH‐dehydrogenases and succinate dehydrogenase, respectively. Reduced coenzyme Q is oxidized by complex III, or cytochrome *c* reductase, which transfers these electrons onto cytochrome *c*, a soluble electron carrier in the intermembrane space. Cytochrome *c* is oxidized by the final complex of the mitochondrial respiratory chain, complex IV or cytochrome *c* oxidase. Depending on the organisms, the energy from electron transfer can be used to translocate protons at NADH reductase, complex III and complex IV. The established proton motive force is then used to power ATP synthases that phosphorylate ADP with inorganic phosphate.

Hence, mitochondrial OXPHOS is a highly valuable cellular machinery. Its evolutionary past from the machinery of the bacterial endosymbiont is clearly visible in the conservation of its principle enzymatic mechanisms. However, the complexes themselves have been structurally remodeled and now contain many more protein subunits than in their bacterial ancestors. One important feature of eukaryotic evolution was the transfer of most of the genes encoding mitochondrial localized proteins to the nuclear DNA. However, a small number of core components of the OXPHOS can be found in the mitochondrial DNA (mtDNA) as a souvenir of the bacterial origin of mitochondria (Neupert, 2016). The suite of conserved genes encoded in mtDNA reflects the need to express these within mitochondria, where they are synthesized by mitochondrial ribosomes (mitoribosomes) and co‐translationally inserted into the mitochondrial inner membrane (Jung et al., 2024). The protein insertase Oxa1 binds, supported by the auxiliary proteins Mba1 and Mrx15, to the mitochondrial ribosome to allow tight coupling of protein synthesis and membrane integration (Jia et al., 2003; Möller‐Hergt et al., 2018; Ott et al., 2006; Szyrach et al., 2003). Most mitochondrial encoded proteins are highly hydrophobic core subunits of OXPHOS. Their hydrophobicity would jeopardize their faithful transport to mitochondria if they were expressed from nuclear genes and synthesized in the cytoplasm (Björkholm et al., 2017; Claros et al., 1995; von Heijne, 1986). At the same time, their sequences cannot be easily changed to allow such transport, as they are optimized for complex electron and proton transfer reactions (Shimada et al., 2026). Hence, these genes are functionally trapped within mitochondria, necessitating the maintenance of an organellar genetic system that involves almost a quarter of the number of mitochondrial proteins (Morgenstern et al., 2017).

The biogenesis of OXPHOS therefore requires the coordinated expression of many different proteins (Couvillion et al., 2016), which derive from two separate genetic systems. Moreover, many proteins need to fold, acquire redox co‐factors for electron transfer, and assemble into high molecular weight complexes, which further involve dedicated assembly factors and quality control systems. In this review, we will highlight recent findings showing how mitochondrial OXPHOS complexes are assembled and maintained to ensure efficient energy conversion in eukaryotic cells.

## 2 SPATIAL ORGANIZATION OF OXPHOS BIOGENESIS IN THE INNER MEMBRANE

**FIGURE 2:**
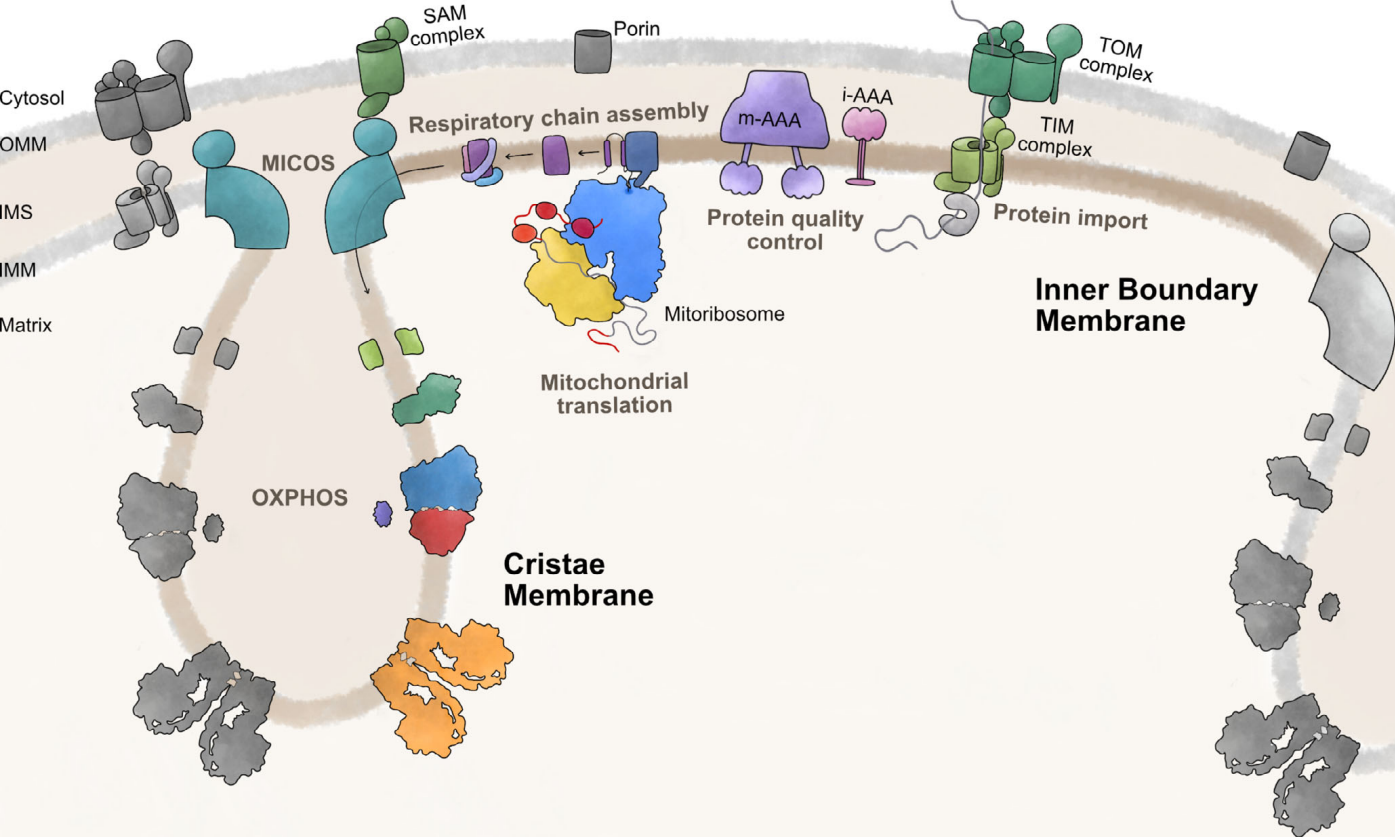
Spatial organization of OXPHOS biogenesis in the inner membrane. Mitochondria contain two membrane systems, the outer mitochondrial membrane (OMM) and the inner mitochondrial membrane (IMM). They create two soluble compartments, the intermembrane space (IMS) and the matrix. Moreover, the IMM is compartmentalized. Here, the inner boundary membrane (which opposes the OMM) is the site of early respiratory chain assembly, while the cristae membranes are the sites of oxidative phosphorylation and the sites of ATP synthase assembly. The MICOS complexes are positioned at cristae junctions and possibly control the composition of both subcompartments. OMM: outer mitochondrial membrane; IMS: intermembrane space; IMM: inner mitochondrial membrane.

Mitochondria have, as a remnant of their evolutionary origin from alpha‐proteobacteria, two membranes, an outer membrane exposed to the cytosol and an inner membrane containing the OXPHOS complexes. Moreover, the inner membrane is folded into invaginations termed cristae, leading to the establishment of subcompartments, namely the inner boundary membrane that opposes the outer membrane, and the cristae membranes that protrude into the matrix (Figure 2). The inner boundary membrane plays a dedicated role in OXPHOS biogenesis, particularly as a site of respiratory chain biogenesis (Stoldt et al., 2018; Toth et al., 2020; Vogel et al., 2006; Werner & Neupert, 1972). The cristae membranes, in turn, are the place of oxidative phosphorylation, where ATP synthase oligomers and respiratory supercomplexes are enriched as also evidenced by cryo‐EM tomography (Davies et al., 2011; Waltz et al., 2025). While initial work suggested that the cristae could help in concentrating protons in their lumen to support proton gradient‐driven ATP synthesis, recent work failed to demonstrate a substantially acidified intra‐cristae space (Rieger et al., 2021; Toth et al., 2020). Instead, it appears that the local enrichment of respiratory chain enzymes and the ATP synthase allows for a tight functional coupling between both activities via local proton transfer (Toth et al., 2020). Nevertheless, the enrichment of active OXPHOS complexes in cristae membranes and their biogenesis sites in the inner boundary membrane requires transport or other sorts of separating both processes to establish subcompartments with divergent functions (Figure 2).

The import of proteins with a cleavable mitochondrial targeting signal (MTS) into the matrix or intermembrane space occurs at TOM‐TIM23 supercomplexes (Figure 2) (Chacinska et al., 2003, 2010), connecting the outer membrane and the inner boundary membrane (Schleyer & Neupert, 1985). Newly imported respiratory chain subunits containing transmembrane segments are inserted into the inner boundary membrane through lateral release at the level of the TIM23 complex (Fielden et al., 2023; van der Laan et al., 2007). Likewise, translation of mitochondrially encoded respiratory chain subunits occurs preferentially at the inner boundary membrane, as are processes occurring during early stages of their assembly (Stoldt et al., 2018). In contrast, synthesis of ATP synthase subunits and their assembly is concentrated in cristae membranes (Stoldt et al., 2018). It is worth noting that most of the TM‐containing subunits of ATP synthase are encoded in mtDNA and do not need to be imported (Jung et al., 2024). Instead, most of the imported ATP synthase subunits are soluble proteins in the matrix. It is hence conceivable that the assembly of respiratory complexes occurs predominantly in the inner boundary membrane because it is here where most of their membrane‐bound subunits are inserted. Moreover, many catalytic subunits of respiratory complexes require the acquisition of redox co‐factors and phospholipids. Possibly, their biogenesis is also concentrated to the inner boundary membrane.

Respiratory chain biogenesis requires a set of distinct folding reactions, redox co‐factor insertion and the establishment of protein–protein contacts in the growing complex. Protein proximity mapping has demonstrated that many of these processes are concentrated around mitoribosomes synthesizing respiratory chain subunits, revealing the existence of early OXPHOS assembly hubs (Kohler, Carlström, et al., 2023; Singh et al., 2020). These hubs could increase the efficiency of assembly by providing a specialized environment where assembly steps are spatially coordinated. How could such subcompartments be established? The biogenesis of cristae is executed by two machineries, ATP synthase through oligomerization (Blum et al., 2019; Paumard et al., 2002) and the activity of the MICOS complex (Daumke & van der Laan, 2025). How the different protein compositions of both subcompartments in the membrane are established and maintained is currently not known. The MICOS complex is positioned at cristae junctions, where cristae membranes bud off from the inner boundary membrane (Figure 2). Placed in this position, MICOS could act as a gate to determine which membrane proteins and complexes can diffuse into cristae membranes. Such a scenario would be in line with data showing that distinct, late stages of complex III and IV assembly could occur close to MICOS (Colina‐Tenorio et al., 2026; Zerbes et al., 2025), so that respiratory complexes upon completion of their assembly are allowed to distribute to the cristae compartment. Likewise, complexome profiling identified a large assembly of lipids and membrane proteins, termed MIMAS (Horten et al., 2024), which could allow maintaining a specific composition of the inner boundary membrane due to its size that would impair spontaneous diffusion into the cristae membranes. The composition of MIMAS with predominantly mitochondrial carrier proteins exchanging molecules between the matrix and the cytosol would be in line with such a function. Moreover, the formation of MIMAS depends on specific lipids (Horten et al., 2024). It is therefore plausible that a specific lipid composition would allow establishing patches within the inner membrane into which specific membrane proteins could partition into.

## 3 BIOGENESIS, IMPORT/SYNTHESIS AND ASSEMBLY OF COMPLEX III

Mitochondrial protein complexes containing subunits derived from nuclear and mitochondrial genomes share one common aspect in their biogenesis, namely that their assembly is highly complex. In baker's yeast, mtDNA encoded proteins include cytochrome *b* (Cyt*b*) from respiratory complex III, Cox1, Cox2, and Cox3 from complex IV, Atp6, Atp8, and Atp9 from the ATP synthase and Var1, a subunit of the mitochondrial ribosome (Figure 1). In the following, we will use the biogenesis of yeast complex III as an example to highlight the complexity of mechanisms that have evolved to assemble OXPHOS complexes in mitochondria.

Mitochondrial complex III, also known as the *bc*_1_ complex or cytochrome *c* reductase, plays a central role in the respiratory chain. It receives electrons from the mobile, hydrophobic electron carrier coenzyme Q to transfer these electrons to cytochrome *c*, a soluble protein of the mitochondrial intermembrane space. To transfer these electrons and convert their chemical energy into proton translocation, complex III contains two pathways for electron transport, which depend on the lining of specific amino acids in its subunits and redox cofactors that can attract and donate electrons (Hunte et al., 2000). Likewise, protons need to be translocated over the inner mitochondrial membrane to establish a membrane potential. These electron and proton transfer pathways are found in three catalytic subunits: Cyt*b*, carrying two heme *b* molecules; cytochrome *c*_1_, containing a covalently bound heme; and the Rieske protein, with its iron–sulfur cluster. In addition to these catalytic subunits, complex III contains seven other structural subunits that are important to stabilize the catalytic subunits and keep them correctly arranged (Ndi et al., 2018). As in the ancestral complex, the mitochondrial complex III is an obligatory dimer. In steady‐state conditions, the dimer of complex III interacts in yeast mitochondria with either one or two copies of complex IV to form respiratory supercomplexes (Hartley et al., 2019; Rathore et al., 2019). These supercomplexes facilitate electron transfer by offering a preferential path for cytochrome *c* diffusion between both complexes' cytochrome *c* binding sites (Berndtsson et al., 2020; Kohler, Barrientos, et al., 2023; Moe et al., 2021).

**FIGURE 3:**
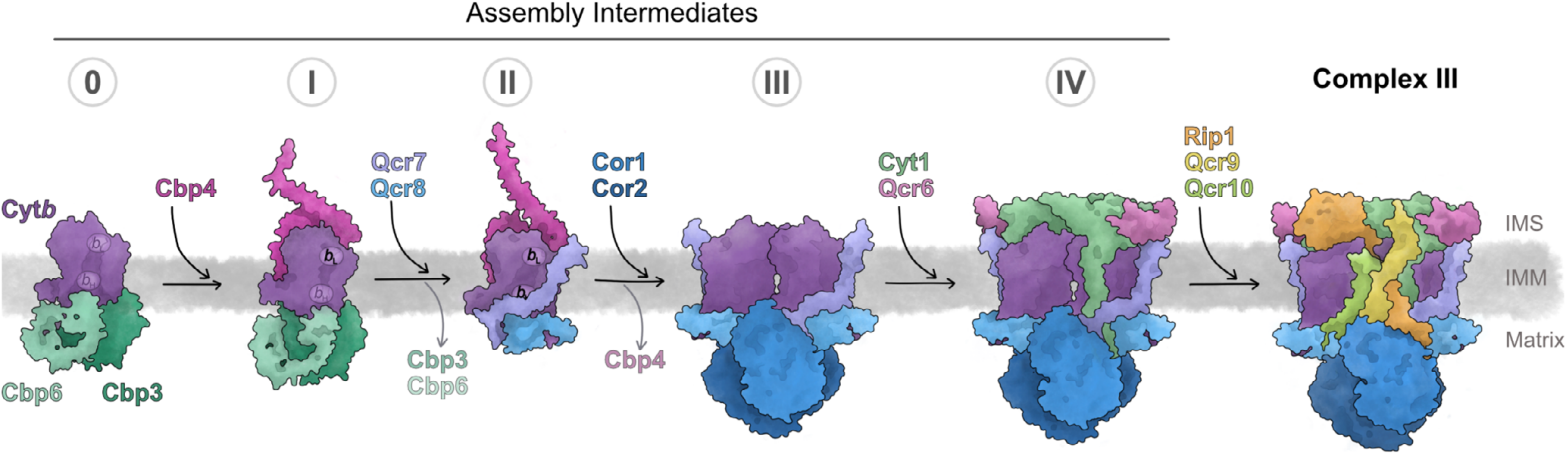
Biogenesis, import/synthesis and assembly of complex III. Respiratory complex III in mitochondria assembles through distinct steps in the inner mitochondrial membrane. These steps are defined by the engagement of assembly factors to stabilize the forming complex. At discrete points during the assembly, they leave the complex and are replaced by structural or catalytic subunits. See text for details. Models were generated according to published work (Berndtsson et al., 2020; Carlström & Ott, 2024). IMM, inner mitochondrial membrane; IMS, intermembrane space; IMS, intermembrane space.

De novo assembly of complex III occurs through distinct steps (Figure 3). The mitochondrial encoded Cyt*b* is the nucleation of assembly. Cyt*b* is synthesized by mitochondrial ribosomes. Its specific assembly chaperone, composed of the highly conserved Cbp3–Cbp6 dimer (Dieckmann & Tzagoloff, 1985; Liang et al., 2022; Wu & Tzagoloff, 1989), binds to the mitoribosomal tunnel exit (Gruschke et al., 2011) to allow for an efficient interaction with nascent Cyt*b*, a contact that is established once its first four transmembrane (TM) helices have been inserted into the inner membrane (Carlström et al., 2025; Carlström & Ott, 2024). For this, Cbp3–Cbp6 has a designated binding site that brings the TM helices of Cyt*b* into the correct spatial arrangement (Carlström & Ott, 2024; Ndi et al., 2019). These four TM helices form a bundle, into which two heme *b* molecules are inserted through coordination by two pairs of axial histidines. How these hemes are inserted is mechanistically not resolved and it is unknown whether there are specific delivery pathways for heme or whether heme biosynthesis and insertion are spatially linked to facilitate diffusion of heme into Cyt*b*. What is clear is that the first heme *b* to get inserted is the one found close to the intermembrane space, denoted *b*_L_, followed by insertion of heme *b*_H_, which binds close to the matrix side of the four‐helix bundle (Hildenbeutel et al., 2014). The order of Cyt*b* hemylation in mitochondria thereby follows the same sequence of events during hemylation as the bacterial homolog (Yun et al., 1991), reflecting their ancestry. However, hemylation of heme *b*_L_ leads in mitochondria to the recruitment of the assembly factor Cbp4 (homolog of human UQCC3 (Wanschers et al., 2014)) to stabilize the acquired heme and to form intermediate I of complex III assembly (Hildenbeutel et al., 2014). This intermediate can act as a reservoir of Cyt*b* ready to assemble and is used for mitochondrial translational regulation (see below).

To progress from intermediate I to II (Figure 3), the assembly factor Cbp3–Cbp6 is released upon hemylation of the *b*_H_ site, after which the two nuclear encoded subunits Qcr7 and Qcr8 bind (Gruschke et al., 2012; Hildenbeutel et al., 2014). Indeed, Qcr7 and Cbp3 share a common binding site on the four‐helix bundle of Cyt*b*, indicating that one function of Qcr7 (and presumably Qcr8) might be to stabilize the bound heme *b*_H_ during and after assembly (Carlström & Ott, 2024). Cbp4 is released from Cyt*b* when it progresses from intermediate II to intermediate III. Interestingly, the TM of Cbp4 binds to Cyt*b* at the dimer interface to the adjacent copy of Cyt*b* (Carlström & Ott, 2024). It is therefore likely that in addition to stabilizing heme *b*_L_ during early steps of assembly, binding of Cbp4 to intermediate I and II might also inhibit the premature interaction between two copies of Cyt*b*. In turn, the release of Cbp4 from Cyt*b* could therefore promote the first steps in the dimerization of complex III.

The next steps of complex III assembly are less well‐resolved, presumably due to various protein–protein interactions that are established between newly imported structural subunits that might be able to occur independently of other, non‐adjacent interactions (Stephan & Ott, 2020). The next stable assembly intermediate that can accumulate is termed intermediate IV, which, in addition to Cyt*b*, Qcr7, and Qcr8, also contains the matrix hetero‐tetramer Cor1–Cor2, cytochrome *c*_1_, and its binding protein Qcr6 (Zara et al., 2009). Through this, two catalytic subunits are brought together into a dimeric form of the assembling complex III (Figure 3), but enzymatic activity is first gained upon incorporation of the last catalytic subunit, the Rieske iron–sulfur protein (Rip1).

Cytochrome *c*_1_ is a protein carrying a covalently attached heme at the N‐terminus, while a C‐terminal TM anchors it to the inner membrane. Biogenesis of cytochrome *c*_1_ is complex, as the protein carries an MTS at the N‐terminus followed by a TM segment. Insertion of this TM into the inner mitochondrial membrane followed by insertion of the C‐terminal TM obtains the correct topology, followed by proteolytic cleavage and removal of the first TM (Arnold et al., 1998). This pre‐mature form of cytochrome *c*_1_ interacts with cytochrome *c*_1_ heme lyase that covalently attaches a molecule of heme (Nicholson et al., 1989). Next, additional N‐terminal processing occurs to generate the mature cytochrome *c*_1_ that is ready to be assembled. Once assembled, it is stabilized on the complex by binding of Qcr6, a soluble protein of the intermembrane space.

The final stages of complex III assembly act on intermediate IV and lead to complex III enzymatic activation, owing to the incorporation of the last catalytic subunit, the Rieske iron–sulfur protein, Rip1 (Figure 3). Rip1 is fully imported into the matrix (Hartl et al., 1986), where it receives its 2Fe–2S cluster by the mitochondrial iron–sulfur cluster assembly machinery (Adam et al., 2006). Because the 2Fe–2S cluster of Rip1 is located in the IMS in the assembled complex, but mounted in the matrix, this domain needs to be translocated in a fully folded form over the inner membrane. The analogous reaction is carried out by the TAT‐translocation system in bacteria, plants and some protists, a machinery that is absent from mitochondria of animals and fungi (Frain et al., 2019; Schäfer et al., 2020). Instead, mitochondria contain a dedicated translocase, the Bcs1 ATPase (Wagener et al., 2011) that is necessary for Rip1 biogenesis (Nobrega et al., 1992). Bcs1 is a member of the widespread family of AAA‐ATPases that play divergent roles in protein metabolism. Typically, these proteins form oligomers that use the concerted action of their associated ATPases to provoke conformational changes. In the case of Bcs1, seven identical subunits form a heptameric structure embedded in the inner mitochondrial membrane (Kater et al., 2020; Tang et al., 2020). Binding of the 2Fe–2S containing Rip1 to the nucleotide‐free Bcs1 induces a closure of the structure, followed by ATP binding to the seven subunits and enclosure of Rip1 (Rosales‐Hernandez et al., 2025). Concerted ATP hydrolysis then induces a conformational change accompanied by opening of the internal cavity and the release of the bound Rip1 protein into the IMS. Once translocated, Rip1 assembles with intermediate IV, an interaction stabilized by Qcr9 and Qcr10. The timing of interaction between complex III and complex IV to form respiratory supercomplexes is unknown. The complexes interact through binding of Cor1 to Cox5 (Berndtsson et al., 2020). Because Cor1 is part of intermediate IV, it would be possible that this intermediate already interacts with complex IV, but such a complex has not yet been described, suggesting that supercomplex formation occurs first between fully assembled complexes III and IV.

## 4 GENETIC REGULATION OF OXPHOS BIOGENESIS

Due to the genetic sources of their subunits, de novo biogenesis of OXPHOS complexes has yet another complication, namely the question of how to coordinate expression of these subunits from two separated genetic systems. This is particularly important during metabolic reprogramming when OXPHOS capacity needs to be increased. The dynamics of metabolic reprogramming can clearly be visualized in baker's yeast, which can readily switch its metabolism from fermentation to respiration, and back (Olivares‐Marin et al., 2018). The necessity to quickly achieve this metabolic reprogramming in competition with other microorganisms has led to the establishment of complex regulatory pathways to minimize waste of resources while allowing rapid growth. Initially, when glucose is rich in the media, yeast cells are growing fast, fueled by energy obtained through fermentation of glucose to ethanol. During this stage of logarithmic growth, respiration is repressed by the presence of glucose, a phenomenon known as “Crabtree‐effect” (De Deken, 1966). Once glucose becomes limited, only ethanol is available as an energy source; therefore, cells undergo major metabolic reprogramming to upregulate expression of OXPHOS enzymes, allowing for the utilization of ethanol through respiration.

**FIGURE 4:**
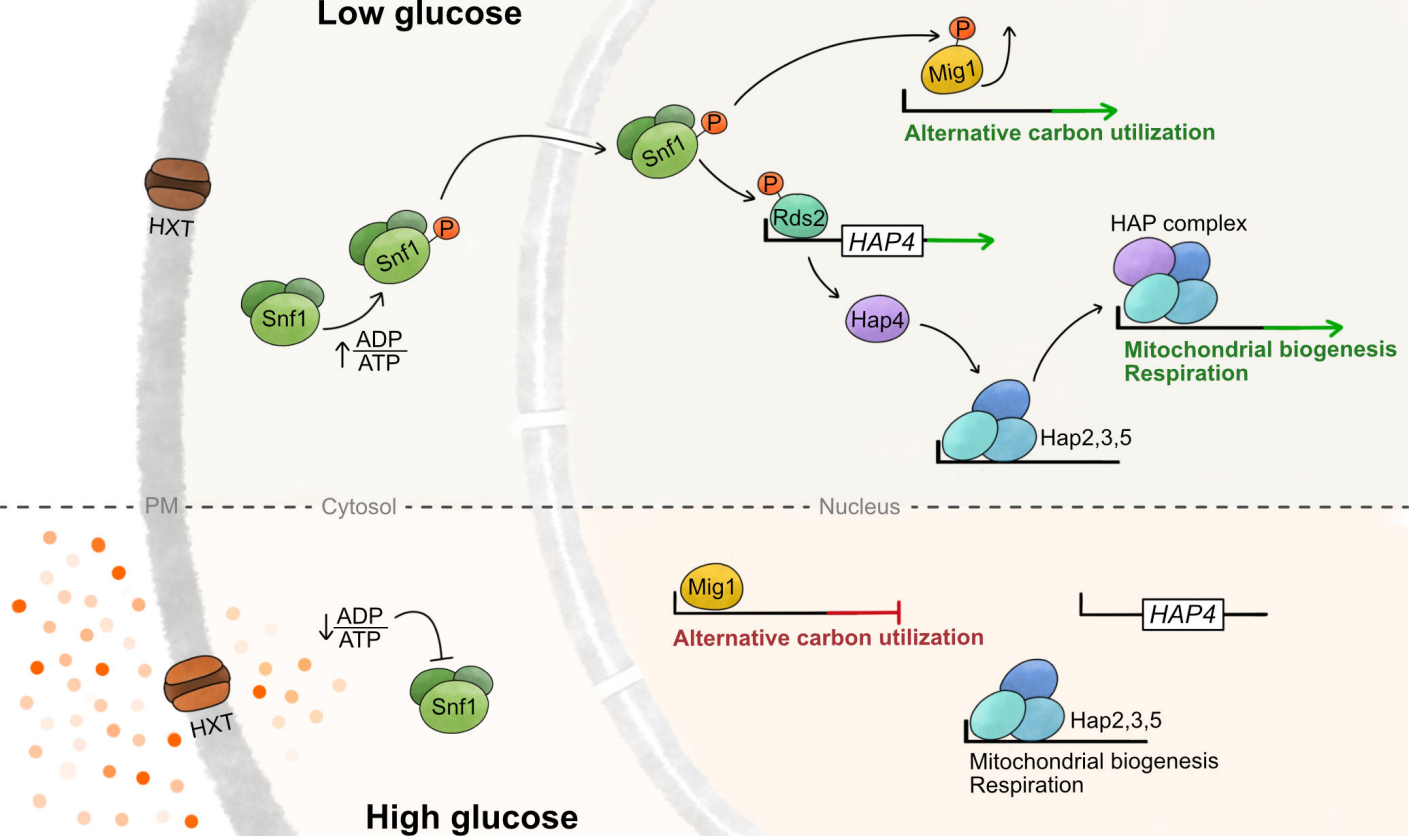
Genetic regulation of nuclear encoded OXPHOS subunits. Glucose fermentation and respiration are two main metabolic pathways in yeast, whose enzymes are reciprocally regulated. When glucose is scarce, Snf1, the AMP‐activated S/T protein kinase (AMPK), gets activated by an increased ADP:ATP ratio. Activated Snf1 gets autophosphorylated and translocates to the nucleus where it phosphorylates transcription factors including Mig1 and Rds2. Phosphorylated Mig1 detaches from the promoters of genes involved in alternative carbon utilization, thereby allowing their expression. Phosphorylated Rds2, in turn, induces expression of HAP4. Hap4 will then join the Hap2,3,5 subcomplex bound to the promoters of respiratory genes, leading to their activation. Under high glucose conditions, a low ADP:ATP ratio will inhibit SNF1's autophosphorylation, hence genes for respiration and utilization of other carbon sources are inactivated, leading to a state known as glucose repression. HXT, hexose transporter; PM, plasma membrane.

The genetic regulation of respiratory genes encoded in nuclear DNA is integrated into cellular metabolism by Snf1, the key regulator of the metabolic effects of glucose on yeast cells (Figure 4). This protein is the catalytic subunit of the highly conserved AMP‐activated S/T protein kinase (AMPK) (Hardie et al., 1998), a multimeric kinase that becomes active upon glucose exhaustion. In yeast, SNF1 is regulated by ADP levels, establishing a direct link to the cell's energetic state (Mayer et al., 2011). Under glucose‐rich conditions, the ADP/ATP ratio in the cytosol is low, hence keeping SNF1 inactive due to autoinhibition (Jiang & Carlson, 1996). When glucose levels decrease (leading to an increase in ADP/ATP levels), the autoinhibition of SNF1 is released and it becomes phosphorylated by several kinases (Sutherland et al., 2003) yielding a fully active AMPK that will induce the expression of glucose‐repressed genes. Most of the transcriptional repression during glucose growth occurs via Rgt1 and Mig1 as the transcription factors that promote the expression of glucose‐related genes, while repressing the transcription of genes involved in the metabolism of other carbon sources including mitochondrial related genes, respectively (Broach, 2012). During the shift to respiration, SNF1 activates downstream transcriptional regulators like Cat8, Sip4, Adr1 and Rds2 that will induce the metabolic reprogramming (Soontorngun et al., 2012; Sutherland et al., 2003). Mitochondrial genes are typically controlled by the Heme Activator Protein (HAP) complex, which is regulated by Rds2 and, thus, SNF1. Active Rds2 binds to the promoter of Hap4, the activator subunit of the HAP complex, promoting its expression, which in turn will induce transcription of nuclear encoded respiratory subunits, genes involved in mitochondrial translation as well as genes required for mitochondrial protein import and dynamics (Buschlen et al., 2003; Soontorngun et al., 2007). Molecularly, Hap4 binds the DNA‐binding subunits (Hap2, Hap3, and Hap5) to form the HAP complex, which promotes gene transcription by binding to the specific sequence motifs present in the upstream activation sites of mitochondrial genes. This interaction of the HAP complex with promotors of mitochondrial genes induces their transcription and the subsequent synthesis of many mitochondrial destined proteins in the cytoplasm, from where they need to be imported into mitochondria (Buschlen et al., 2003).

In addition to gene regulatory mechanisms controlling nuclear genes, yeast also has mechanisms to control mitochondrial gene expression at the level of the organelle. Central to this organelle‐specific regulation are dedicated translational activators. Discovered early during the mapping of genes necessary for respiration (Cabral & Schatz, 1978; Ebner et al., 1973), later work demonstrated that they are RNA‐binding proteins that mediate translation of one specific mitochondrial encoded mRNA (Costanzo & Fox, 1990; Ott et al., 2016). Genetic work established that the translational activators act on the 5′‐untranslated region (5′‐UTR) of the mRNAs (Costanzo & Fox, 1993; Sanchirico et al., 1998), and that mutations in translational activators leading to respiratory growth defects can be compensated for by mutations in proteins of the small ribosomal subunit (Haffter et al., 1991; McMullin et al., 1990). More specifically, these mitoribosomal proteins are mostly yeast‐specific proteins that are found adjacent to the mRNA channel exit in the small ribosomal subunit where they form a V‐type canyon‐like structure (Desai et al., 2017). Indeed, structures of yeast mitoribosomes with translational activators for *ATP9* or *ATP8* mRNAs bound to this ribosomal domain revealed how the translational activators help in mediating translation of their client mRNA (Bridgers et al., 2025). Through binding the 5′‐UTR to mostly secondary structural elements and specific locations on the small mitoribosomal subunits, translational activators align the mRNAs on the ribosome for translation initiation (Bridgers et al., 2025). Because translational activators are rate‐limiting for translation of their client mRNA (Steele et al., 1996), their expression from nuclear genes can be used to indirectly regulate protein synthesis of mitochondrial encoded proteins.

**FIGURE 5:**
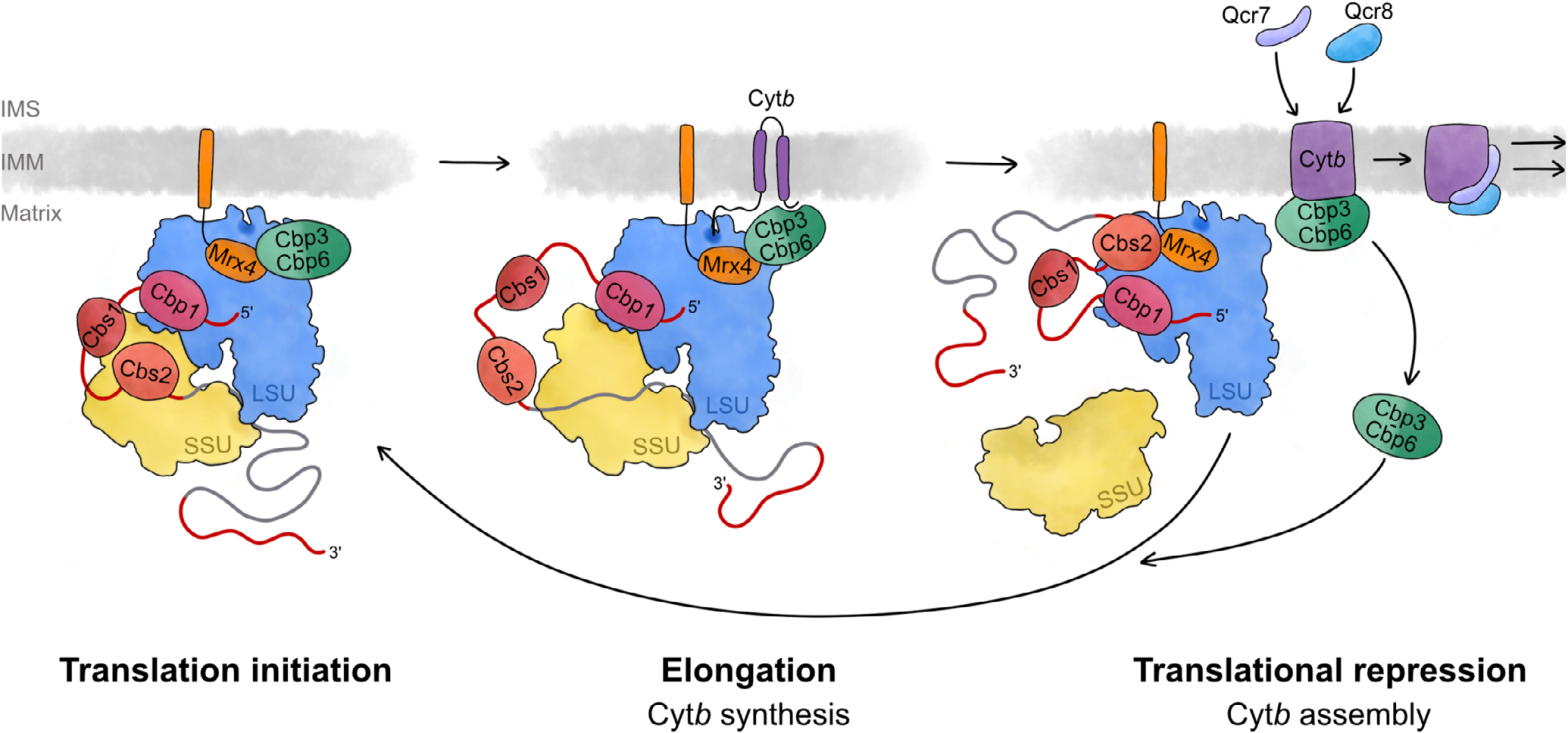
The translational feedback loop adjusting the levels of cytochrome *b* synthesis. Cytochrome *b* (Cyt*b*) is encoded in the mitochondrial DNA and synthesized by mitoribosomes. Its synthesis is regulated by a translational feedback loop. To activate translation of the Cyt*b* encoding mRNA, the assembly factor Cbp3–Cbp6 interacts with Mrx4 at the mitoribosomal tunnel exit on the large subunit (LSU). This allows the mRNA with bound translational activators Cbs1 and Cbs2 to interact with the small ribosomal subunit (SSU) for translation initiation. During elongation, the translational activators are released from the small subunit and Cbp3–Cbp6 establishes interactions with the nascent Cyt*b*. Upon completion of Cyt*b* synthesis, the full‐length protein interacts with Cbp3–Cbp6 and leaves the mitoribosome to form intermediate I of the complex III assembly (Figure 3). Now, Mrx4 at the mitoribosomal tunnel exit is free to interact with Cbs2 and the bound Cyt*b*‐encoding mRNA, thereby inhibiting its interaction with the small subunit and preventing translation initiation. Once Cbp3–Cbp6 is released from the assembling Cyt*b*, it can compete off Cbs2 from Mrx4 and by this induce a new round of Cyt*b* synthesis. IMM, inner mitochondrial membrane; IMS, intermembrane space.

For the case of complex III, expression of the only mitochondrial encoded subunit, Cyt*b*, is controlled by four translational activators (Ott et al., 2016). Interestingly, the expression of Cyt*b* is controlled also by a specific translational feedback loop, which adjusts the levels of Cyt*b* synthesis inside mitochondria depending on the efficiency by which it assembles (Figure 5). Cyt*b*‐encoding mRNA contains a large 5′‐UTR, which is bound by three translational activators, namely Cbp1, Cbs1, and Cbs2. The molecular function of Cbp1 is to bind near the 5′ end of the mRNA to protect it from degradation (Dieckmann et al., 1984). Cbs1 and Cbs2 are two canonical translational activators (Rödel, 1986) that bind the mRNA channel exit of the mitoribosome and to specific motifs within the 5′‐UTR of the Cyt*b*‐encoding mRNA to promote its translation initiation (Islas‐Osuna et al., 2003; Salvatori et al., 2020; Singh et al., 2020). Importantly, the access of the translational activators to the mRNA channel is regulated, a feature that eventually determines rates of Cyt*b* synthesis. The key regulatory event here is whether the assembly factor Cbp3–Cbp6 can bind to the polypeptide tunnel exit in the large mitoribosomal subunit (Salvatori et al., 2020). Binding of Cbp3–Cbp6 to the tunnel exit can only occur when the complex is not bound to Cyt*b*, a state that occurs when the assembling Cyt*b* is bound to nuclear encoded subunits (Figure 3), hence reporting ongoing complex III assembly (Gruschke et al., 2011; Gruschke et al., 2012). Under these conditions, more Cyt*b* should be produced. Binding of empty Cbp3–Cbp6 to the tunnel exit includes an interaction with Mrx4, which binds to the Cyt*b* binding site within the complex (Carlström et al., 2025; Ndi et al., 2019). Mrx4 is a ligand of the mitoribosomal tunnel exit, which can either bind to Cbp3–Cbp6 or to the translational activator Cbs2 (Carlström et al., 2025). Hence, when Cbp3–Cbp6 is free, it binds to Mrx4 at the tunnel exit, but once binding to fully synthesized Cyt*b*, it leaves the ribosome, allowing Cbs2 with complexed mRNA to bind to Mrx4 (Carlström et al., 2025). This places the Cyt*b*‐encoding mRNA in a location where it cannot be used for translation; hence production of more Cyt*b* is inhibited. However, once Cyt*b* is released from the Cbp3–Cbp6 complex and bound by Qcr7 and Qcr8, Cbp3–Cbp6 can again bind to Mrx4. This then releases Cbs2‐bound mRNA from Mrx4, upon which new rounds of Cyt*b* synthesis can be initiated. Similar feedback mechanisms operate during the biogenesis of Cox1 (Barrientos et al., 2004; Mick et al., 2007; Perez‐Martinez et al., 2003), the central subunit of complex IV, Atp6 (Rak & Tzagoloff, 2009), the proton channel of ATP synthase, and Var1 (Seshadri et al., 2020), a component of the small mitoribosomal subunit. These feedback control loops avoid overproduction of the mitochondrial‐encoded membrane subunits, which is wasteful and could jeopardize the integrity of the inner mitochondrial membrane and the essential membrane potential.

## 5 TURNOVER AND RECYCLING FOR MAINTENANCE

An important part of early assembly hubs is proteases, which are positioned proximal to the OXPHOS assembly lines, mitoribosomes and the import machinery (Figure 2) (Kohler, Carlström, et al., 2023; Singh et al., 2020). Specifically, the highly conserved prohibitin‐mAAA supercomplex plays a dedicated role in degrading mitochondrial encoded subunits that are either produced in excess or fail to assemble for other reasons (Kohler, Carlström, et al., 2023). The prohibitin ring forms a cage that houses, in analogy to the bacterial system (Ma et al., 2022), in its interior the m‐AAA protease, in yeast a heterohexamer composed of alternating Yta10 and Yta12 subunits (Arlt et al., 1996). This protease contains an AAA‐ATPase that extracts proteins from the membrane and feeds the linearized polypeptide into the proteolytic chamber for degradation. The functional significance of the prohibitin ring for proteolysis is yet unknown. Given that its absence accelerates protein turnover by the m‐AAA protease (Kohler, Carlström, et al., 2023; Steglich et al., 1999), it is conceivable that prohibitin acts as a molecular filter to allow only specific proteins, likely unfolded and non‐assembled membrane proteins, to get access to the protease. This would be in line with observations that Cyt*b*, when produced in excess in the absence of Cbp3–Cbp6, gains increased access to the prohibitin‐m‐AAA complex for degradation (Kohler, Carlström, et al., 2023). However, other mechanisms are also possible, for example, an impact on the local lipid environment (Osman et al., 2009) affecting the organization of the assembly lines in the inner boundary membrane or phospholipid‐dependent folding of newly synthesized proteins.

Membrane protein degradation is also very important for metabolic remodeling and turnover of unfunctional or damaged OXPHOS complexes (MacVicar et al., 2019; Ohba et al., 2020). In the cytosol, specific systems exist for the identification and regulated removal of proteins and their complexes through the ubiquitin‐proteasome system. In mitochondrial membranes, similar directed degradation systems do not exist. Induced degradation of mitochondrial inner membrane proteins can be achieved in two ways. First, the metalloprotease Oma1 can be activated upon reduction of the mitochondrial membrane potential (Ehses et al., 2009; Head et al., 2009). Oma1 then cleaves substrate proteins at specific sites in their transmembrane segments, thereby inducing their unfolding and subsequent degradation by AAA‐connected proteases (Kaser et al., 2003). Secondly, prolonged protein unfolding, possibly due to radical‐induced damage, can turn proteins into substrates for m‐AAA and i‐AAA proteases that recognize misfolded proteins (Graef et al., 2007; Kaser et al., 2003; Leonhard et al., 1999). Such mechanisms could play important roles in disassembling OXPHOS complexes containing damaged subunits. By degrading the damaged subunits, the remaining subunits could be recycled in the assembly process, leading to an exchange of the damaged subunit with a new functional copy. In case there is no new functional copy provided by the gene expression machinery, for example, during metabolic reprogramming, the remaining subunits fail to be stabilized and are hence degraded. Such a scenario is in line with observation from SILAC‐labeling and quantitative proteomics that revealed that individual subunits of OXPHOS complex have substantially different half‐lives, even if they are part of the same complex (Christiano et al., 2014). These more labile subunits are often small and found in the periphery of the complexes, suggesting that they might dissociate more easily from the assembled complex to then be replaced by new proteins. The new proteins will then join the assembly processes toward fully functional respiratory chain complexes. The combination of balanced de novo assembly pathways and quality control‐regulated recycling of subunits allows the maintenance of a functional OXPHOS system.

## 6 CONCLUDING REMARKS

The biogenesis of mitochondrial OXPHOS complexes is a sophisticated process, relying on mechanisms to regulate expression of mitochondrial and nuclear encoded subunits, their transport, folding and maturation through incorporation of redox co‐factors. Moreover, these proteins need to be assembled into multi‐subunit complexes, which in turn are distributed to specific locations within the mitochondrial inner membrane. Here, we used the biogenesis of complex III as an example to demonstrate shared and specific solutions to these challenging biogenesis pathways. Within the sequence of events leading to a fully assembled complex, many steps are not fully mechanistically resolved, which is true also for the biogenesis of the other OXPHOS complexes. One of the most intriguing questions relates to the problem of how individually imported subunits are merged into a single growing OXPHOS complex during assembly. It is currently unknown whether these interactions occur in a random, collision‐based fashion or whether they are spatially orchestrated, which would be in line with the identification of early assembly hubs (Singh et al., 2020). If the latter scenario is the case, how are these hubs organized and how are precursor proteins directed into these sites? Are there different populations of import sites that attract different sets of precursor proteins? Moreover, the coordinated *de‐novo* assembly, recycling and removal of OXPHOS complexes as it happens during steady state conditions or during metabolic remodeling should require further levels of coordination between all these activities (Ghifari et al., 2025). Because the mitochondrial OXPHOS system is a highly valuable machinery, its dysfunction leads to severe human diseases. It is an important task for future research to fill these knowledge gaps to allow a mechanistical understanding of how OXPHOS is established and maintained.

## AUTHOR CONTRIBUTIONS

**Andreas Carlström:** Writing – review and editing; visualization; conceptualization. **Carmela Vazquez‐Calvo:** Visualization; writing – review and editing. **Martin Ott:** Conceptualization; writing – original draft; writing – review and editing; resources; project administration; funding acquisition; supervision.

## Data Availability

The data that support the findings of this study are available from the corresponding author upon reasonable request.
